# Factors influencing interest in recreational sports participation and its rural-urban disparity

**DOI:** 10.1371/journal.pone.0178052

**Published:** 2017-05-25

**Authors:** Chiehfeng Chen, Liang-Ting Tsai, Ching-Feng Lin, Chun-Ching Huang, Yao-Tsung Chang, Ruey-Yu Chen, Shu-Yu Lyu

**Affiliations:** 1 Division of Plastic Surgery, Department of Surgery, Evidence-Based Medicine Center, Wan Fang Hospital, Taipei Medical University, Taipei, Taiwan; 2 Department of Public Health, School of Medicine, College of Medicine, Cochrane Taiwan, Taipei Medical University, Taipei, Taiwan; 3 Taiwan Marine Education Center, National Taiwan Ocean University, Keelung City, Taiwan; 4 School of Public Health, Taipei Medical University, Taipei, Taiwan; 5 Keelung Hospital, Ministry of Health and Welfare, Keelung City, Taiwan; 6 Department of Public Health, Fu Jen Catholic University, New Taipei City, Taiwan; 7 Department of Exercise and Health Science, National Taipei University of Nursing and Health Sciences, Taipei, Taiwan; 8 Department of Leisure Industry and Health Promotion, National Taipei University of Nursing and Health Sciences, Taipei, Taiwan; Texas Technical University Health Sciences Center, UNITED STATES

## Abstract

**Objectives:**

Recreational sports are important leisure activities. However, most studies investigating barrier factors and motivation for participation in recreational sports have been limited to specific areas (e.g., a city or school) or demographic groups (e.g., adolescents). Therefore, this study set out to gain a more comprehensive understanding of the behavioral and socioeconomic factors influencing interest in recreational sports participation in Taiwan, as well as to evaluate the effect of any urban-rural divide.

**Methods:**

This study analyzed data collected by the “Taiwan Social Change Survey” (program five, wave 3) “Leisure Life” questionnaire. We used hierarchical linear modeling to assess respondent interest in recreational sports participation and evaluated the influence of behavioral factors, socioeconomic factors, and residence location (urban/rural).

**Results:**

Of the 2,146 participants in this study, 50.3% were male, and the average age was 43.9 years. Location of residence (urban/rural) accounted for 35.3% of the variation in interest in recreational sports participation, while the remaining 64.7% came from the individual level. Participants who lived in rural settings were less interested in recreational sports than their urban counterparts. Gender, educational attainment, participation frequency, health-motivated interest, and appearance-motivated interest were also associated with interest in recreational sports participation.

**Conclusions:**

Different communication strategies may be needed to effectively reach different demographic groups. We suggest that future public health campaigns aiming to increase recreational sports participation include tailored interventions and messages to effectively encourage leisure physical activities among all, regardless of demographic boundaries.

## Introduction

Recreational sports play an important role in our leisure activities and have significant influences on human physiology and psychology. Physiologically, exercise has tangible benefits both in promoting health and reducing mortality rates. Regular and moderate recreational sports not only reduce the risk of diabetes, cardiovascular disease, and cancer [1–3], but are also reflected in a variety of mortality rates through associations with health indicators such as BMI. [4]. Research in 2011 by Wen et al. indicated that in Taiwan, regardless of gender or cardiovascular disease status, individuals who engage in moderate-intensity exercises such as fast walking for 15 minutes daily, or 92 minutes weekly, benefit from 14% reduced mortality rates and a three-year extension in average lifespan compared with those who do not exercise at all. If the duration of exercise is increased another 15 minutes daily, the mortality rate drops by another 4% [5]. From a psychological point of view, recreational sports are social activities, and encourage interaction between different social groups. Recreational sports not only increase physical and mental health, but also reduce psychological pressure, increase self-affirmation, promote positive cognitive and psychological development, and lower risk of depressive symptoms [6].

The Taiwanese public has been encouraged to increase their participation in recreational sports for decades, however the results to date have not been outstanding. Thus, further investigations are needed to ascertain how to promote participation most effectively. Many factors influence participatory behavior and motivation to participate in recreational sports, including demographic characteristics, such as gender, age, educational attainment, and socioeconomic status [7, 8]. Job characteristics, such as job control, working hours, and job security, also affect the frequency and willingness to spend time on leisure activities [9, 10]. Certainly, one's health status is also influential. For example, heart disease patients often worry that exercise may not be suitable for them, thereby reducing their participation in leisure activities [11]. The local environment is another consideration, and the space available for recreational sports, the distance to facilities, and quality of the equipment all naturally impact willingness to participate [12]. These issues moreover demonstrate significant rural-urban disparities [13, 14].

The main focus of this study was the rural-urban disparity in recreational sports participation. A study by Wang et al. indicated that urban and rural areas have different and unique living spaces that engender different and unique lifestyles, with differences in lifestyle naturally being reflected in activity participation [15]. That study further indicated that a rural-urban disparity has existed in leisure activities since 1990. Previously, urbanized Taiwanese tended to participate in consumption-satisfaction leisure activities such as shopping. However, by the year 2000 the trend in leisure activities had shifted to more traditionally rural activities such as walking, hiking, camping, and fishing. Other studies from Western countries have also found that rural residents generally participate in low-intensity activities orientated toward agriculture and housework, while urban residents generally participate in primarily moderate/high-intensity recreational sports [16, 17]. These differences demonstrate the necessity of taking the rural-urban disparity into consideration in the promotion of recreational sports.

Currently, research on the Taiwanese public’s motivational and obstructive factors regarding recreational sports participation is both sparse and limited to specific areas (e.g., a county or school) and groups (e.g., teenagers). Although research targeting specific regions, groups, and occupations makes special contributions to the literature, it inevitably leads to findings that fail to have valid inferences that can be generalized to other environments. Thus, the focus of this study was to try to analyze two different levels of data together. Traditional regression analyses generally miss the interactions of different context variables due to the inherent difficulties in dealing with the cross-level information arising from nested features in survey data. Therefore, this study set out to utilize a hierarchical linear model (HLM) which allowed us to aim the focus of the study at individual-level variables (i.e., the public’s interest in recreational sports participation) and forecast and analyze different levels of explanatory variables at the individual level (e.g., an individual’s educational level) and the overall level of urban and rural environments (e.g., employment status rates), thus achieving stratification predictions. Using HLM, we explored differences in recreational sports interest between urban and rural residences to fill in the current lack of a comprehensive study and examine the impact of different rural-urban disparities on the public’s interest in recreational sports in Taiwan from the perspective of a cross-level analysis.

Therefore, in addition to exploring the influence of behavioral factors and socioeconomic status characteristics on interest in recreational sports, this study also explored differences in recreational sports participation interest between urban and rural residences through a cross-level analysis as a reference for future policy-makers and public health interventions.

## Materials and methods

### Sample and data collection

This project analyzed data collected by the Taiwan Social Change Survey, Phase 5, Wave 3 "Casual Living" survey. The study was approved by the Joint Institutional Review Board of Taipei Medical University (N201607049). All the data can be downloaded freely from the following website: (https://srda.sinica.edu.tw/group/scigview/1/2).

The design of the questionnaire was based on trends in Taiwanese social changes to explore the leisure status of Taiwanese society. This questionnaire included the International Social Survey Programme’s (ISSP) 2007 survey "Leisure Time and Sports Questionnaire” (LTSQ) title set as the basis for international comparisons [18]. The sampling method of the social change investigation included population density, educational attainment, percentage of the population over the age of 65, percentage of the population aged 15–64 years, industrially employed percentage of the population, commercially employed percentage of the population and other indicators. We divided Taiwan’s townships into seven strata and used a three-stage stratified equal probability proportional to size sampling (PPS) to extract 2,147 respondents. A sampling analysis with the population parameter (goodness of fit) displayed a population characteristic distribution that was consistent with the population parameter data, thus indicating that the findings are nationally representative.

### Measures

This study focused on factors involved in recreational sports. This study defined participation in recreational sports as simply engaging in physical activities. “What is your degree of interest in recreational sports participation?” was the dependent variable, and the options were classified into two categories: "not interested/not very interested" and "somewhat interested/quite interested/very interested."

This study’s purposes were to (a) assess behavioral factors and socioeconomic status features that affect the public’s interest in recreational sports, and (2) estimate the percentage of variation in the public’s interest in recreational sports participation that can be explained by urban and rural residence status. Therefore, this study contained two different levels of analysis and discussion. The first level belongs to the individual level, and the second belongs to the overall level of urban-rural residences.

### Variables

This study’s main dependent variable was whether a subject was interested in participating in recreational sports. In addition to the dependent variable, we considered two independent stratified variables which are described here.

#### Level one: Individual level

Individual-level independent variables may change with different population characteristics and socioeconomic status standards, thus influencing interest in recreational sports participation. Thus, individual behavioral factors are the most influential factors in recreational sports interest. Meanwhile, our study also integrated the aforementioned studies and selected variables containing gender, educational attainment, participation frequency, health-motivated interest, and appearance-motivated interest. The educational level variable included junior high school and below, senior high school, and junior college and above. Frequency of recreational sports participation was dichotomized to daily and not daily. The dichotomized physical and health-motivated interest variable reflects whether a subject’s interest in recreational sports participation was for the benefit of their physical or mental health, whereas the dichotomized appearance-motivated interest variable reflects whether a subject’s interest in recreational sports participation was for the benefit of their physical appearance or figure.

#### Level two: Overall level of urban-rural residences

According to Warner-Smith and Brown's research [19], certain employment opportunities, income instability, and other factors may result in extreme limitations on recreational sports participation. In Taiwan, employment opportunities and income varied in different urban-rural residences. We therefore selected independent variables including employment status, average monthly income, and other variables, and explored whether differences in urban-rural residences indirectly affected public interest in recreational sports participation.

### Statistical analyses

This study used an HLM analysis to assess the impact of the explanatory variation sources of interest in recreational sports participation between urban and rural residences. This allowed us to explain the sources of variation and estimate the impact of the two levels of explanatory variables, as well as assess whether there were cross-level interaction effects. In our multi-level analyses, we used the Taiwan Social Change Survey database to process two-level nested pairing, after proper weighting, to present the overall situation of the public’s interest in recreational sports participation in Taiwan.

In the multi-level analysis, this project used a null model (model 1) to understand differences besides those of the urban-rural residences, a random coefficient model (model 2), an average number of outcome variables model (model 3), and a full model (model 4), to gradually explore the impact of variables of all levels on interest in recreational sports participation.

## Results

### Descriptive statistics

In this investigation the study population consisted of individuals aged over 18 years in Taiwan (born before December 31, 1977). The actual survey sample consisted of 2,147 individuals, but after deducting one participant who lacked data for physical activity 2,146 samples remained. A summary of the descriptive statistical analysis is shown in Table 1 along with accompanying homogeneity of proportions tests to investigate differences between groups. Of the participants interested in the recreational sports participation, 48.7% were female, 58.0% were aged above 40 years, 39.0% completed junior college or above, 50.4% lived in areas with a high degree of urbanization, and only 28.0% participated in daily leisure physical activities. Of the interested participants, 88.8% agreed that their interest was motivated by a concern for physical or mental health, and 47.1% agreed that their interest was motivated by a concern for their figure and physical appearance.

**Table 1:** Descriptive statistics of participant characteristics.

Variables	Interest in recreational sports participation		
	some interested/quite interested/very interest (n = 1,642) n(%) = 76.5%	not interested/ not much interested (n = 504) n(%) = 23.5%	p-value
Gender			0.067
Female	799(48.7)	269(53.4)	
Male	843(51.3)	235(46.6)	
Age			<0.001
18–29	381(23.2)	86(17.1)	
30–39	309(18.8)	79(15.7)	
40–49	315(19.2)	108(21.4)	
50–59	307(18.7)	86(17.1)	
≧60	330(20.1)	145(28.8)	
Degree of urbanization			<0.001
High(core urban region/general urban region)	828(50.4)	201(39.9)	
Medium(emerging cities and townships /traditional industrial sector cities and townships)	595(36.2)	184(36.5)	
Low(general townships and villages/rural areas)	219(13.3)	119(23.6)	
Educational level			<0.001
Junior high school or below	523(31.9)	307(60.9)	
Senior high school	478(29.1)	111(22.0)	
Junior college or above	641(39.0)	86(17.1)	
Participation frequency			<0.001
Daily	459(28.0)	29(5.8)	
Not daily	1,183(72.0)	475(94.2)	
Health-motivated interest^a^			<0.001
Very important/Important	1,446(88.8)	319(70.3)	
A little important/Not important	183(11.2)	135(29.7)	
Appearance-motivated interest^b^			<0.001
Very important/Important	761(47.1)	129(28.2)	
A little important/Not important	855(52.9)	328(71.8)	

^a^: number of missing data = 63

^b^: number of missing data = 73

### Individual and rural-urban level influences

The multilevel analytical results of the public’s interest in recreational sports participation are shown in Table 2. The focus of this study was the proportion of the variance (intraclass correlation, ICC) in recreational sports participation within the rural-urban level. This value was calculated as follows:

$$\begin{array}{l} \text{rural-urban level ICC}\\ \;\frac{\tau_{00}}{\tau_{00+}\sigma^{2}}=\frac{0.088}{0.088+0.1613}=0.3530 \end{array}$$

**Table 2:** Effects of individual and rural-urban disparity factors on interest in recreational sports participation.

	Null model (model 1)	Model 2	Model 3	Full Model (model 4)
Intercept	0.786 (p = 0.017)	0.784 (p = 0.013)	0.787 (p = 0.015)	0.784 (p = 0.012)
Individual-level factor				
Male gender		0.038 (p = 0.018)		0.038 (p = 0.018)
Educational attainment		0.114 (p = 0.013)		0.112 (p = 0.012)
Frequent participation		0.240 (p = 0.028)		0.241 (p = 0.028)
Health-motivated interest		0.185 (p = 0.031)		0.184 (p = 0.032)
Appearance-motivated interest		0.0699 (p = 0.017)		0.070 (p = 0.017)
Rural-urban disparity level factors				
Degree of urbanization			-0.024 (p = 0.017)	-0.019 (p = 0.009)
Income			0.006 (p = 0.008)	-0.003 (p = 0.007)
Variance components				
Rural-urban disparity level variance (τ_00_)	0.088	0.047	0.076	0.041
Individual level variance (σ ^2^)	0.161	0.139	0.161	0.139

The results indicated that the individual level and rural-urban level accounted for 35.30% and 64.70% of the total variance in recreational sports participation, respectively.

Table 2 also contains the coefficients (and p-values in parentheses) of the dependent variables that were significant in the model. These coefficients represent the influence of the dependent variable on interest in recreational sports participation. This is depicted on a scale ranging between 0 and 1, with 0 representing not interested/not very interested, and 1 representing somewhat interested/quite interested/very interested. In the case of a dichotomous variable, the coefficient can be interpreted as the influence of the positive aspect of the variable on this scale with the negative aspect being the reference (e.g., health-motivated interest produces the effect in reference to not having a health-motivated interest). The influence of the coefficient in polytomous variables is interpreted as moving up one level with the baseline level as the reference category (e.g., senior high school is up one level from the reference category junior high school or below). In both cases, the coefficient describes the influence of the variable in terms of how much more an individual with that variable would be on the scale from 0 to 1, or how much more likely they would be to be interested in recreational sports.

The random coefficient model (model 2) included individual-level variables under the original structure of model 1 and revealed that male gender (0.038), increasing educational attainment (0.114), frequent participation (0.240), health-motivated interest (0.185), appearance-motivated interest (0.069), and other variables significantly (p < 0.05) affected interest in recreational sports participation.

The average number of outcome variables model (model 3) added rural-urban level variables under the original structure of model 1. The results of this model showed that the degree of urbanization significantly (*p* < 0.05) affected interest in recreational sports participation. The more rural the area where the respondent lived, the less interest they expressed in recreational sports participation.

The results of the full model (model 4), which included individual-level and rural-urban level variables, were the same as models 2 and 3.

## Discussion

The results of this study showed that 35.3% of the variance regarding interest in recreational sports participation stemmed from rural-urban status, with the rest being explained by individual level factors. The more rural the area where a respondent lived, the less interest they expressed in recreational sports participation. Other factors that affected interest in recreational sports participation were gender, educational attainment, health-motivated interest, and appearance-motivated interest).

Our results are consistent with other studies which pointed out that rural populations are less willing to participate in recreational sports [14, 16, 20], that body image will affect one's willingness to exercise [21], that there is a gender disparity in exercise interest [22, 23].

The types of exercise rural and urban residents engage in differ considerably [24]. Exercise among rural residents is largely related to farming chores and other work, so most rural resident activity is low-intensity and high-duration, while urban resident activity generally focuses on more moderate/high-intensity exercises. Furthermore, while the former is a natural consequence of work, the latter arises due to a specific desire to engage in exercise behaviors. Moreover, previous studies have observed that in urban and rural area parks, there is a rural-urban disparity in park user's exercise behaviors [25]. Although the usage of country parks is higher than city parks, users in the city are more likely to engage in exercise than users in the countryside.

Some prior studies detected factors that we were unable to include in our analysis. These include differences in type of work [16, 26], availability of sports facilities [14, 27, 28], security of the living environment [13], parental diet and exercise behaviors [29], and musculoskeletal pain [30]. As these factors were not included in the analysis they may be possible explanations for differences in urban/rural recreational sports participation interest detected in this study.

The promotion of exercise-related policies is critical to the future of Taiwan. Taiwan is entering an era with a rapidly aging population and an increased prevalence of chronic diseases caused by a lack of exercise. These factors will continue to substantially increase the burden on the healthcare system. However, not only should factors regarding interest in recreational sports be explored, but also actual exercise behaviors.

In light of this, we offer a few suggestions for future research and policies. First, future research should investigate actual exercise behaviors and differences in the self-efficacy of urban and rural residents to determine effective focal points for interventions and exercise-promotion policies. Second, exercise-promoting policies should distinguish the rural-urban disparity, and promote exercise according to urban and rural characteristics. Third, a longitudinal study should be used to verify the results of exercise behavior promotion according to the rural-urban disparity. Promoting exercise behaviors and improving the public’s regular exercise behaviors are important for suppressing the prevalence of chronic diseases due to an aging population as well as for managing the burden on the healthcare system. Fourth, a discussion of exercise self-efficacy is necessary [31], especially with regard to the rural-urban disparity. Since exercise is not considered a necessity by Taiwanese social norms, most Taiwanese prioritize their responsibilities for work and family, thus compressing available time for recreational sports and reducing the willingness to exercise. According to health behavior theory, these factors affect self-efficacy and decision-making in exercise behavior, and also of course, exercise consistency [32, 33].

Finally, this study had several limitations. First, the study used a cross-sectional analysis and therefore cannot effectively investigate causal relationships between the rural-urban disparity and actual exercise behaviors. Second, the study did not investigate relationships between health behavior theory indicators in exercise patterns in urban and rural areas, nor did the study deal with actual exercise behaviors. Thus, we are unable to discern the exact nature of the relationships between the investigated variables and actual exercise participation in this study. However, this is the first study to use improved statistical methodology to investigate rural-urban disparities in the public’s interest in recreational sports participation. These findings can be used as references for future research methods and for investigating the rural-urban disparity in exercise behaviors.

## Conclusions

This study found that the more rural a location the less interested its residents were in recreational sports participation. We also found gender, educational attainment, health-motivated interest, and appearance-motivated interest to be important factors affecting recreational sports participation. Recommendations for the future promotion of exercise behavior should use tailored methods based on the rural-urban disparity to effectively promote recreational sports participation.
